# Supplementary material for: 1,4-Naphthoquinone (CNN1) Induces Apoptosis through DNA Damage and Promotes Upregulation of H2AFX in Leukemia Multidrug Resistant Cell Line
Source: Int J Mol Sci. 2022 Jul 23;23(15):8105. doi: 10.3390/ijms23158105 (PMC9330061; doi:10.3390/ijms23158105)
Supplement: Supplementary file 1 [file ijms-23-08105-s001.zip › ijms-1805063-supplementary.pdf]

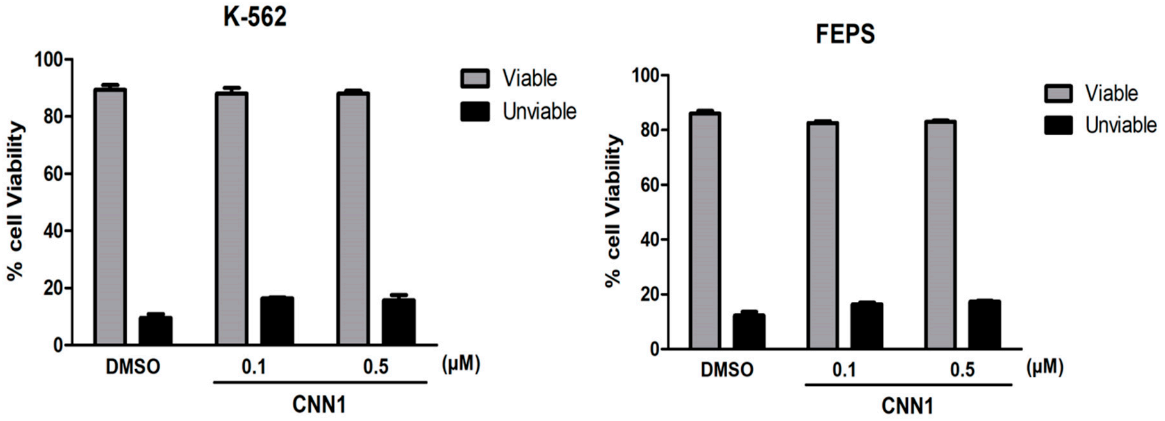

Figure S1: Trypan blue assay of K-562 and FEPS after 18h of treatment with **CNN1**. The bars represent the mean ± standard error of three independent experiments in triplicate. There was no statistically significant difference in cell viability comparing the negative control with treated group by ANOVA followed by Tukey test.

Table S1. Sequence of oligonucleotides used for RT-qPCR.

Gene	Sequence (5’ – 3‘)	NCBI reference sequence
H2AFX	F- ACCAGCACAAGTCGGTTA	NM_002105.3
	R - AAGGTTCTAGTCGTGGAAGG	
ACTB[1]	F- CTGGAACGGTGAAGGTGACA	NM_001101.5
	R- AAGGGACTTCCTGTAACAACGCA	

[1]Actin Beta (ACTB) gene was used as the endogenous control.
